# Supplementary material for: Content-rich biological network constructed by mining PubMed abstracts
Source: BMC Bioinformatics. 2004 Oct 8;5:147. doi: 10.1186/1471-2105-5-147 (PMC528731; doi:10.1186/1471-2105-5-147)
Supplement: Additional File 2 — The original results of the above study (non-essential files are deleted to keep the file size under the limit set by BMC bioinformatics). [file 1471-2105-5-147-S2.bz2 › chilibotAdditionalFile2/dip05/146ID8895367E520/html/IGF1R_PTPN11.html]

 


 **IGF1R** and **PTPN11** 
  
Found 8 abstracts in PubMed, retrieved 05.  
 

 What does Google say? 
 PDF only 
| .edu only 

---

**Interactive relationship** (e.g. stimulation, inhibition, etc)

**Non-interactive relationship** (e.g. studied together, co-existance, homology, etc.)

- Tyrosine phosphorylation of the beta 3 subunit of the alpha V beta 3 Integrin is required for membrane association of the tyrosine phosphatase SHP 2  [ **PTPN11** ]  and its further recruitment to the insulin like growth factor I receptor  [ **IGF1R** ] .  Ref: 12791772 Mol Endocrinol< MedlineTA>Mol Endocrinol,
- Regulation of insulin like growth factor I receptor  [ **IGF1R** ]  dephosphorylation by SHPS 1 and the tyrosine phosphatase SHP 2  [ **PTPN11** ] .  Ref: 11779860 J Biol Chem, 2002
